# The views of physicians and nurses on the potentials of an electronic assessment system for recognizing the needs of patients in palliative care

**DOI:** 10.1186/s12904-020-00554-9

**Published:** 2020-04-04

**Authors:** Natalia Radionova, Gerhild Becker, Regine Mayer-Steinacker, Deniz Gencer, Monika A. Rieger, Christine Preiser

**Affiliations:** 1 Institute for Occupational Medicine, Social Medicine and Health Services Research, University Hospital Tuebingen, Wilhelmstraße 27, D-72074 Tuebingen, Germany; 2 Clinic for Palliative Care, Faculty of Medicine, Medical Centre University of Freiburg, University of Freiburg, Freiburg, Germany; 3 Department of Internal Medicine III, University Hospital of Ulm, Ulm, Germany; 4 Department of Medicine III, Medical Faculty Mannheim, University Medical Centre Mannheim, Heidelberg University, Mannheim, Germany; 5 Core Facility Health Services Research, University Hospital Tuebingen, Tuebingen, Germany

**Keywords:** Electronic symptom assessment, symptoms & symptom management, user acceptance, qualitative research.

## Abstract

**Objectives:**

Patients in oncological and palliative care (PC) often have complex needs, which require a comprehensive treatment approach. The assessment of patient-reported outcomes (PROs) has been shown to improve identification of patient needs and foster adjustment of treatment. This study explores occupational routines, attitudes and expectations of physicians and nurses with regards to a planned electronic assessment system of PROs.

**Methods:**

Ten physicians and nine nurses from various PC settings in Southern Germany were interviewed. The interviews were analysed with qualitative content analysis.

**Results:**

The interviewees were sceptical about the quality of data generated through a patient self-assessment system. They criticised the rigidity of the electronic assessment questionnaire, which the interviewees noted may not fit the profile of all palliative patients. They feared the loss of personal contact between medical staff and patients and favoured in-person conversation and on-site observations on site over the potential system. Interviewees saw potential in being able to discover unseen needs from some patients. Interviewees evaluated the system positively in the case that the system served to broadly orient care plans without affecting or reducing the patient-caregiver relationship.

**Conclusions:**

A significant portion of the results touch upon the symbolic acceptance of the suggested system, which stands for an increasing standardisation and technisation of medicine where interpersonal contact and the professional expertise are marginalized. The study results can provide insight for processes and communication in the run-up to and during the implementation of electronic assessment systems.

## Background

As a result of longer life expectancy and medical progress, the number of people living with an incurable, fatal disease is increasing [1]. Those affected are often facing complex physical, psychological and social problems, which can be caused both by the disease itself and by its treatment. In oncological and PC settings, physicians and nurses are confronted with the challenge of recognising the diversity of problems faced by patients and incorporating possible solutions and measures into treatment planning.

It has been shown that patient assessments in regards of their needs by means of questionnaires can not only benefit the patient, but also help further research on PC treatment effectiveness [2, 3]. These questionnaires are filled in by the patients themselves or, if necessary, by their representatives. The assessment of PROs in oncological or palliative settings can lead to 1. increased attention by physicians and/or caregivers, 2. better identification of needs [3–5], for example with regard to psychosocial problems [6–8], 3. improve physician-patient communication, 4. adjustment of treatment [3, 4, 9–12]. Previous studies have shown that early needs assessment and the ensuing treatment tailored to patient needs can result in a noticeable improvement in the quality of life of patients and the course of the disease [13]. However, the effects of PROs assessment on the actual health-related quality of life are still not clearly understood [3, 4, 9, 14]. Although it is still unclear to what extent PROs are integrated into practice in daily patient care routines [4], studies suggest that integration is rather low in general practice [15–18] as well as PC [19]. Factors preventing the integration of PROs in practice include: medical staff being unconvinced by PROs assessment benefits; long, incomprehensible or inappropriate questionnaires; lack of training for medical staff to facilitate the interpretation and implementation of the assessment results [4, 14].

Studies suggest that a meaningful integration of PROs into clinical routine can be fostered by an electronic assessment [4]. Various electronic screening instruments and systems for patient self-assessment in oncology and PC have been developed and their implementation tested in English and German-speaking countries (e.g. [20–22]). However, paper-and-pencil procedures still dominate the assessment of PROs [3] and electronic assessments of PROs are not very widespread in standard care yet [5]. Feasibility and implementation studies show numerous factors that have to be considered for a successful application and acceptance of electronic assessment systems. Both physicians and nurses [23] as well as patients need support and contact persons for operating the devices and programs [24]. Medical staff users must see the benefits of a system [25] but react negatively if they feel limited in their scope of action [23], if a system causes more efforts than decreasing them [5] or is not well-integrated into workflows [26]. Local routines and knowledge cultures should also be considered in order to understand how a system will fit into them [5].

According to implementation theory, several factors affect the successful implementation of new health care innovations such as the innovation itself, the target group of professionals, the patients, the setting and the methods and strategies of dissemination and implementation [27]. Innovations which are consistent with the professional knowledge of practitioners are more likely to be implemented than other innovations [27]. We have focused on physicians and nurses working in the PC field as a target group in this qualitative study (duration: 09/2016–04/2019). As potential professional users and gate-keepers to the innovation, so their knowledge, skills, opinions, attitudes, values, routines, and/or personalities can affect implementation. The study is part of the collaborative project MySUPPORT (07/2016–12/2019). In MySUPPORT, structures and processes for the routine recording and clinical use of PROs in different palliative and oncological care contexts are to be implemented and evaluated. Ideally, the data collection and the presentation of the results will be done via a web-based application. In the Tuebingen subproject, PC professionals were interviewed before the implementation of the PROs assessment system in the settings of the project partners in order to inform the implementation process. The patient perspective will be assessed in the final evaluation of the collaborative project.

The subproject had two general research questions: first, what do physicians and nursing staff of various PC settings expect from the planned assessment system? Second, which professional self-images can be derived from the data? Thus, we broaden the perspective beyond questions of feasibility or acceptance of PROs and electronic assessment systems (cf. [28]). We will focus in this article on three subquestions regarding the expectations of physicians and nurses: What do we learn about current methods of patient needs assessment? How do the interviewees estimate the quality of the PROs? Which potentials and limitations do they see in the electronic assessment of PROs?

## Methods

### Design

The basis of the study was an investigation of expectations of physicians and nurses ahead of the development and implementation of the electronic assessment system. As such, we decided to use semi-structured expert interviews [29]. The interviewer used a prepared interview guide to structure the interviews and phrased ad hoc follow-up questions to ensure openness at the same time [30]. We addressed the interviewees as professionals with a high competence in explicating their expertise [29] and manifest meaning [31]. Given the expected level of explication and our main interest in themes, we used qualitative content analysis [31] to answer the first general research question.

### Sample and participants

Nineteen semi-structured face-to-face interviews were conducted with physicians and nursing staff from various PC settings in Baden-Wuerttemberg (Germany).

As all project partners were based in Baden-Wuerttemberg, data was collected from only this province. Since the community is rather small, we will need to provide rather broad information about the sample in order to not expose our interviewees. A broad variety of interviewees were selected in order to represent the heterogeneity of the professionals and settings in PC as well as to include all settings of potential implementation. In Germany, PC is provided in inpatient settings (hospices, specialised PC units in hospitals, nursing homes) and in outpatient settings (specialised outpatient palliative care service (SAPV), GP care, outpatient nursing service providers) [32].

**Fig. 1:**
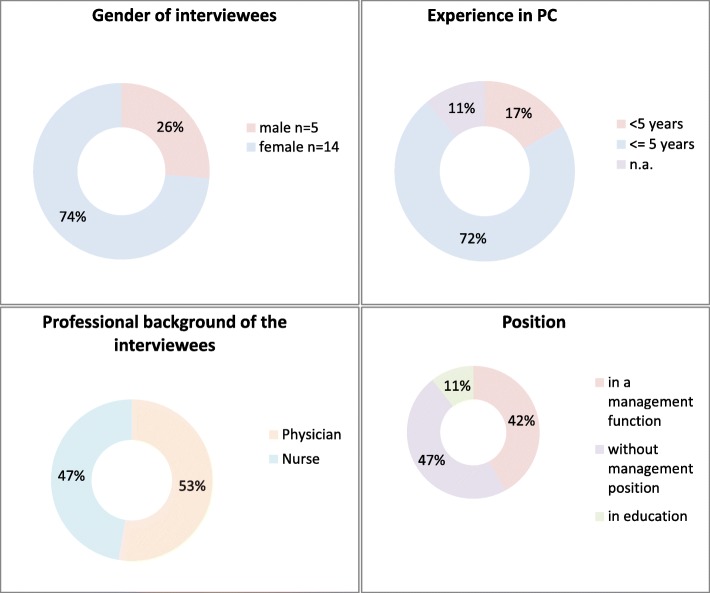
Sample of the study

We aimed to interview experts of different ages and genders coming from medical and nursing professions in inpatient and outpatient PC settings and being at different stages in their careers. The final sample consisted of 10 physicians and nine nurses (see Fig. 1). All main inpatient and outpatient settings for PC were included (see Table 1). The physicians and outpatient nursing services were recruited by letter. Inpatient nurses were recruited according to the snowball sampling technique in the facilities of the respective physicians. We finished data collection when we had reached a heterogeneous sample but no new themes arose from further interviews [31].

**Table 1:** Sample of the study

SETTING		Professionals
Inpatient care	Hospice	1x physician
		3x nurses
	Clinical setting	5x physicians
		3x nurses
Outpatient care	General practice	4x GPs
	Nursing service provider	1x nurse
	Specialised outpatient care (SAPV)	2x nurses

### Methods of data collection

The interviews were conducted at a place chosen by the interviewees, which was in all cases at or close to their workplaces [28]. All interviews were conducted by NR, a sociologist with no background in PC and no professional ties to the interviewees. As such, NR was addressed several times as a sales representative of the planned system and had to clarify her role as researcher. Consequently, she communicated more clearly that she was not involved into the implementation process. The interviews lasted 30 to 55 min, with an average duration of 40 min. All interviewees were informed about confidential handling of their personal data beforehand. A declaration of consent was signed.

The interviews had two main sections (all topics see Table 2): In the first part, interviewees were asked about their professional background and current processes to assess patients’ needs within their respective setting. Then, a planned electronic assessment system was described as following *“Imagine if there were an electronic screening system that could be used to assess patient needs. Possible complaints and individual needs of patients receiving palliative care could be determined in a standardized way. The patients would use this system themselves, for example on a tablet. The data would then be accessible to all caregivers.”* The interviewees were asked about their expectations of such a system with regards to their specific setting. All interviews were audio recorded with a device that had no Wifi or Bluetooth port. They were transcribed by a professional transcription service and then pseudonymised by NR who replaced all names, places and employers in order to protect the interviewees.

**Table 2:** Main topics of the interview guide

**Introduction**	• Professional background. • Setting background, special features of palliative care in setting. • Structures, organization; working conditions; care goals; clientele.
**Current palliative care in own setting**	• Processes of PC in setting. • Needs assessment practices. • Contact/relationship between nurses/physicians and patients.
**Expectations concerning the planned electronic assessment system**	• Needs assessment. • Possibility to use and integration into routines. • Changes in interaction and doctor-patient relationship. • Need to use, benefits. • Barriers to use. • Possible changes on patient side.

### Data analysis

The data material was analysed with the qualitative content analysis according to Schreier [30]. Four heterogeneous interviews were selected to ensure variability and grasp a broad range of topics. Each interview was segmented thematically and each segment was paraphrased and reduced to preliminary codes based on interview themes. Further preliminary codes were deduced from the interview questions. The preliminary codes were brought into a hierarchical order to develop a coding frame that reflected the main thematic dimensions of the material (= main codes) and their content differentiation (= subcodes). A definition and at least one illustrating quote were added to each code. During trial coding, the preliminary coding frame was rechecked with the four interviews and reworked to make distinctions between codes clearer. After this pilot phase, the entire data material was coded with the same coding frame. In order to do so, we integrated the coding frame and all interviews to MAXQDA 11. In a last step, intra-code and inter-code comparison were conducted by NR, CP and the student assistants.

### Reflexivity

Every step of the analysis was carried out simultaneously and independently by at least NR and one of the three student assistants and then discussed jointly to examine consistency [30] and achieve intersubjectivity [31]. Several data sessions with CP helped to focus the analysis. Since NR, CP and the student assistants were sociologists, further meetings with MAR and the whole collaborative project made sure that critical feedback from medical professionals was also included to increase validity. The standards for reporting qualitative research guidelines [33] were applied in this article.

## Results

The following results are presented in the present tense and without indirect speech in order to maintain immediacy. Quotations are presented in italics in the text, indicating the care setting and the professional group of the interviewees. Individual characteristic words of the interviewees are marked with quotation marks. Interestingly, patterns could be found across settings, professions, age and gender. Consequently, it means that interviewees from all groups and settings, but not necessarily all interviewees, have voiced similar aspects, if interviewees are not further specified. The structure of the results does not follow the coding frame, but the structure of the inter-code comparison [30]. We will give a short overview of current practices of patient needs assessment in PC. Then, we will present the interviewees’ perspectives on anticipated data quality before we will elaborate on expected potentials and limitations of the assessment system in the last section.

### Current assessment practices of patients’ needs

We asked interviewees about their current assessment practices in order to understand whether electronic assessment of PROs was already part of their professional routines or not. The interviewees provide a very heterogeneous picture of the current assessment practices and the knowledge and application of standardised assessment methods. The wording in our interviews is accordingly heterogeneous, whereby the term “scales” is repeatedly used as a symbol for standardised and standardising assessment in general. Some of the interviewees from inpatient settings describe the use of various, even standardised, instruments. Interviewees from the hospice make it clear that “scales” are generally of little use in their setting as they value the presence of caregivers over standardised assessments of patients’ needs. All interviewees pick up on (their) knowledge, competences and experience/intuition in order to identify relevant needs. In addition, interviewees expressed that interprofessional cooperation and team meetings produce a more comprehensive and complex picture of the patients among the individual team members. GPs formed the only exception as they saw themselves rather isolated from interprofessional exchange opportunities. All interviewees expressed full satisfaction with their current assessment practices: *“If you have a long-standing palliative care unit with really good staff, not only the nursing staff, all professional groups, everyone must always work together, and everyone sees something different and that gives the overall picture, and I believe that we are really good there […] maybe that is arrogant, but I do believe that we usually already know what the patient needs, also regarding psychosocial care.”* (Inpatient care, nurse 1).

### Estimation of data quality

The interviewees reflect on the possible quality of the PROs collected by the electronic assessment system and consider the use of scales, incomplete or non-valid data. Interviewees criticise that standardised PROs do not adequately reflect the subjective reality of the patients and the complexity of their needs. They also raise the question which subjects can be queried in “scales” at all. This is discussed especially with regards to pain and psychological symptoms. Interviewees expect the questions to be one-dimensional and not doing justice to the multidimensionality of pain or distress. Others assume that at least first tendencies can be explored through standardised questions. For some interviewees, physical well-being can be better expressed in “scales” than psychological well-being. Therefore, psychological well-being can only be addressed in direct interaction: *“Physical needs can be relatively well indicated with the scales, for example ‘How severe is the pain on a scale or the shortness of breath?’ [personal topics] which then occupy one’s mind, are more difficult to draw”* (Inpatient care, physician 3)*.* GPs also consider “scales” to be suitable for asking about spiritual needs.

In general, interviewees assume that not all patients will be able to operate the electronic assessment system due to age and/or illness and/or media affinity. This means that patient data will be available of only a part of all patients. The interviewees expect the PROs to depict the relevant symptoms and mood of the patient at the time of entry. Thus, the data only represent a snapshot of the patient’s actual condition. From the interviewees’ point of view, this makes it more difficult to assess whether the outcomes indicate a short-term acute condition or a stable general condition: *“I trust what I have asked the patients myself more than an assessment, as already mentioned above, which the patient may give in an acute situation in a way that is probably no longer comprehensible afterwards, and which is then available in the evaluation. If I were to confront the patient with this, he would say:” “Well, that was the acute situation, but that has nothing to do with my actual basic feeling*.” (Inpatient care, physician 3).

For interviewees, these results into a standardising and rigid picture of the patients, which does not take possible rapid change of (physical) conditions into account. They criticise that the focus on (dominant) symptoms reduces the view on patients and their individual situations to predefined scales: *“Because you reduce someone to a symptom, a problem, and [ …] the patient may be in pain, the relatives are afraid and worried. And a third person is full of questions and wants to have another discussion about therapy, so to speak. And that diversity is lost there.”* (SAPV, nurse 1).

The interviewees also question the self-assessment of patients and consequently the validity of the PROs. They expect outcomes to be biased, since not all patients will express their needs, for example due to disease and symptom repression or due to a lack of reporting symptoms. Also, not all patients could translate their symptom experience into the queries: *“‘Do you feel depressed?’ and he says ‘no’, and he has not recognised that his sleep disorder is also a form of depression [...]”* (Inpatient care, physician 2). The tendency of some patients to dramatising or strongly reserved self-reporting was mentioned as a further possible bias in response behaviour. The interviewees also point out that the needs assessment system does not allow practitioners to trace back the influence of relatives on patients’ self-assessment-processes and inputs.

### Potentials and limitations of the assessment system

Some of the interviewees saw potential opportunities of the planned electronic assessment system in identifying afflictions. This includes patients and practitioners alike. Interviewees conceive that the queries of the system might stimulate the patients to reflect on their own physical and mental feelings. In addition, physicians see the possibility that the system might reduce patients’ feelings of shame. The recognition of hidden problems might be promoted with regards to some topics and some patient groups: “*Nobody admits gladly that he or she is depressive or something like that. But if he or she can indicate it on a scale with a value and send it off, it is sometimes easier to express than if I had to say it to someone’s face.”* (Inpatient care, physician 5).

According to interviewees, the system provides a chance for practitioners to identify undiscovered needs early on. In this function, it is conceivable for them to use the electronic assessment system in the sense of a basic assessment for palliative needs, especially in non-specialised outpatient settings. They see a potential for prompt problem recognition especially for patient groups with lower contact frequency to physicians and nurses in outpatient care. This applies, for example, to the settings for nursing homes: *“Because sometimes, when we have quite a lot of patients in specialised home care, when people do not report themselves, it happens that none of us will look after them for six weeks at all”* (SAPV, nurse 2).

The interviewees assume that the outcomes can train the practitioners to look at different problem areas. This could concern topics that some physicians classify as subordinate, such as *“shamefaced or fear-laden”* topics (GP 3) or spiritual-needs. Interviewees from inpatient settings suspect that the electronic assessment system could support inexperienced clinical physicians in recognising needs or sensitise inpatient PC staff to the wishes and needs of patients.

All interviewees consider observations and face-to-face interactions as more suitable methods for identifying needs than an electronic assessment system that is filled out in the absence of physicians. Face-to-face interaction with the patients allows them to react immediately, situationally and flexibly according to the condition and wishes of the patients. Even small-talk and trivial topics of conversation provide nurses and physicians alike with additional information for the (holistic) appraisal of the current situation and symptoms. The observations of non-verbal signals such as body language or movement, facial expressions or intonation provide them with further important clues to fully recognise the patients’ conditions and needs. Hidden problems and individual needs behind variables and simple answers can be identified during immediate interaction. *“That’s just the kind of medical skill you’d notice: There’s more to it than the simple ‘no’ or the simple ‘yes’. Otherwise I wouldn’t need to do my work anymore, otherwise I could put an algorithm there, then the patients enter their things and the perfectly proportioned pills are spit out.”* (Inpatient care, physician 1).

Some of the interviewees expect that the application of the electronic assessment system will focus the needs assessment as some questions will be omitted in further conversation, e.g. if the outcomes are unambiguous: *“Sure, if someone indicates zero and has no pain, then you can leave it at that and say that this will not be the problem perhaps.”* (Hospice, nurse 3). Others address the possible consequences of the unchecked acceptance of the collected PROs: *“If the physician just fails to deal with the patient personally, but simply says ‘Oh, no depressions, okay’, well, then it doesn’t go any further at this point.”* (Inpatient care, physician 2).

According to some of the interviewees, the planned electronic assessment system does not provide any fundamentally new information about the needs of patients and is therefore superfluous in routine care. If PROs offer identical findings as a conventional conversation, they represent unnecessary data duplication. Thus, the electronic assessment system would be another burden for the already burdened patients. In their view, the electronic assessment system offers no room to explore patients’ individual concerns and symptoms. At best, PROs do not represent final findings about the condition of the patients, but open up new questions that should always be checked critically in direct interactions with patients: *“You have to look specifically at what the person said and what they want and what they need. And that’s where I have to look. Is now his problem his nausea or is his problem that he simply needs someone to talk to outside of the family or that he simply has to clarify spiritual things with a pastor. And then that also has further effects, then I see what his main problem is and I see: What do I have to work on.”* (Inpatient care, nurse 1).

All interviewees have in common that for them the planned system cannot and must not replace a personal conversation with patients: *“It depends on whether the physician sees this tool as a screening instrument that does not replace the fact that he is nevertheless dealing with the patient, or whether this tool is a kind of substitute for talking to the patient, according to the motto: Have you documented everything completely and collected these and the figures?”* (Inpatient care, physician 1)

## Discussion

The qualitative study was carried out in advance of the planned development of an electronic assessment system. It thus does not address the feasibility or acceptance of existing potential system, but illuminates the perspectives on knowledge cultures and processes on site and how a planned system is anticipated by PC physicians and nurses [5, 28]. Our results show heterogeneous assessment methods being currently applied across the respective settings. Our results indicate that PROs are hardly integrated into patient needs assessment and that patient needs are assessed in face-to-face interaction with the PC professionals. Interviewees questioned the validity and quality of insights collected from PROs. On the one hand the validity of the potential data is doubted, since scepticism prevails over patients’ self-assessments (cf. [21]). This touches upon the general trustworthiness of PROs in the eyes of PC professionals. On the other hand, the planned electronic assessment system is criticised as a form of standardisation of the queries that lead to an incomplete picture of the patient’s situation. Incomplete data records are also expected, since the given type of query would not be accessible to all patients. These reservations apply specifically to the planned electronic assessment system. The results indicate high parallels to another study in which it was also shown that physicians justify their rejection of an electronic screening system via their respective patient collective [19].

When it comes to potentials and limitations of the assessment system, one of the main concerns is the loss of personal contact with the patients. This is expected to have a detrimental effect on the patients in PC to whom interviewees attribute a particularly high need for contact. Interviewees evaluated the system positively in the case that the system served to broadly orient care plans without affecting or reducing the patient-caregiver relationship. As in [19], medical or nursing staff put their own intuition before the possibilities of a standardised needs assessment.

When implementing electronic assessment (systems), it is not so much the patients who are sceptical as the physicians and nurses [26]. The planned electronic assessment system, for example, symbolises an increasing standardisation and technisation of medicine for the occupational groups interviewed, in which the interpersonal contact and the respective professional expertise are marginalised (cf. [19]). Conversely, this suggests that the acceptance of technical healthcare innovations among physicians and nurses depends on whether they recognise their benefits (cf. [25]), whether they feel compromised or supported by the innovation and how well they are prepared for it (cf. [25, 28]).

### Limitations

The qualitative research approach enabled a well-founded exploration of the expectations of potential future users on the medical staff’s side. For ethical reasons, the Tuebingen project dispensed with interviewing patients. The patient perspective was integrated during the evaluation of the implementation at a later stage of the collaborative project. The structure of the collaborative project led to a regional focus on the federal state of Baden-Wuerttemberg. Further perspectives and experiences against the background of regional differences in PC cannot be ruled out. Nevertheless, we managed to achieve a heterogeneous sample in which physicians and nurses of different genders, age groups and experience levels of professional life, as well as different settings of PC were included. As no further substantially new themes arose with additional interviews, we decided the data to be saturated [31] after 19 interviews. This seems rather surprising at first glance, given the heterogeneity of the sample. We assume that this results from the fact that a large part of the expectations stay on the level of symbolic acceptance [34], i.e. fewer practical problems are discussed than the questions of what an electronic assessment of PROs represents for physicians and nurses.

## Conclusion

The study results can provide support for processes in the run-up to and during the implementation of electronic assessment systems. It encourages to reflect during a given implementation process, which aspects of current working routines might be changed or be replaced and what this would mean for the target group. The results could also improve the communication about an innovation. We conclude from our data, that practical challenges regarding technology usage, processes, responsibilities and data protection can be clarified through training and by permanent key persons (cf. [28]). Other topics, such as doubts about data quality or concerns about deterioration in the relationship with patients, require more sound communication (cf. [14]). These require information tailored to the specific setting which emphasises which role the system will have in patient contact, how it will support professional expertise and how it will lead to better PC.

## Data Availability

Data is not available for third parties or full publication. Relevant quotes were pseudonymised in order to protect the study participants.

## Abbreviations

GP: General practitioner
PC: Palliative Care
PRO: Patient-Reported Outcomes
SAPV: German: Spezialisierte Ambulante Palliativversorgung. English: Specialised home care

## References

[1] Bundesministerium für Gesundheit: Nationaler Krebsplan. 2017. https://www.bundesgesundheitsministerium.de/themen/praevention/nationaler-krebsplan/der-nationale-krebsplan-stellt-sich-vor.html. Accessed 31 July 2019.

[2] Bausewein C, Daveson BA, Currow DC, et al. EAPC-White Paper on outcome measurement in palliative care: Improving practice, attaining outcomes and delivering quality services - Recommendations from the European Association for Palliative Care (EAPC) Task Force on Outcome Measurement. Palliat Med. 2016;30(1):6–22. 10.1177/0269216315589898. 10.1177/0269216315589898 26068193

[3] Etkind SN, Daveson BA, Kwok W, et al. Capture, transfer, and feedback of patient-centered outcomes data in palliative care populations: does it make a difference? A systematic review. J Pain Symptom Manage. 2015;49:611–24. 10.1016/j.jpainsymman.2014.07.010. 10.1016/j.jpainsymman.2014.07.010 25135657

[4] Howell D, Molloy S, Wilkinson K, et al. Patient-reported outcomes in routine cancer clinical practice: a scoping review of use, impact on health outcomes, and implementation factors. Ann Oncol 2015;26(9):1846–58. 10.1093/annonc/mdv181. 10.1093/annonc/mdv181 25888610

[5] Johnston BM. Palliative home-based technology from a practitioner’s perspective: benefits and disadvantages. Smart Homecare Technol Telehealth. 2014;(2):121–8. 10.2147/SHTT.S42687.

[6] Koehler M, Hornemann B, Holzner B, et al. Zukunft jetzt – Implementierung eines IT-gestützten Distress-Screenings: Expertenbasierte Konsensempfehlungen zum Einsatz in der onkologischen Routineversorgung. Onkologe. 2017;(23):453–61. 10.1007/s00761-017-0209-7.

[7] Hilarius DL, Kloeg PH, Gundy CM, et al. Use of Health-related Quality-of-Life Assessments in Daily Clinical Oncology Nursing Practice. A Community Hospital-based Intervention Study. Cancer. 2008;113(3):628–37. 10.1002/cncr.23623. 10.1002/cncr.23623 18543317

[8] Detmar SB, Muller MJ, Schornagel JH, et al. Health-Related Quality-of-Life Assessments and Patient-Physician Communication. A Randomized Controlled Trial. JAMA. 2002;288(23):3027–34. 10.1001/jama.288.23.3027. 10.1001/jama.288.23.3027 12479768

[9] Bouazza YB, Chiairi I, Kharbouchi OE, et al. Patient-reported outcome measures (PROMs) in the management of lung cancer: A systematic review. Lung Cancer. 2017;113:140–51. 10.1016/j.lungcan.2017.09.011. 10.1016/j.lungcan.2017.09.011 29110842

[10] Berry DL, Blumenstein BA, Halpenny B, et al. Enhancing patient-provider communication with the electronic self-report assessment for Cancer: a randomized trial. J Clin Oncol. 2011;29(8):1029–35. 10.1200/JCO.2010.30.3909. 10.1200/JCO.2010.30.3909 PMC3068053 21282548

[11] Cleeland CS, Wang XS, Shi Q, et al. Automated Symptom Alerts Reduce Postoperative Symptom Severity After Cancer Surgery: A Randomized Controlled Clinical Trial. J Clin Oncol. 2011;29(8). 10.1200/JCO.2010.29.8315. 10.1200/JCO.2010.29.8315 PMC3068055 21282546

[12] Velikova G, Booth L, Smith AB, et al. Measuring quality of life in routine oncology practice improves communication and patient well-being: a randomized controlled trial. J Clin Oncol. 2004;22(4):714–24. 10.1200/JCO.2004.06.078. 10.1200/JCO.2004.06.078 14966096

[13] Black N, Burke L, Forrest CB, et al. Patient-reported outcomes: pathways to better health, better services, and better societies. Qual Life Res. 2015;25:1103. 10.1007/s11136-015-1168-3. 10.1007/s11136-015-1168-3 26563251

[14] Boyce MB, Browne JP, Greenhalgh J. The experiences of professionals with using information from patient-reported outcome measures to improve the quality of healthcare. BMJ Qual Saf. 2014;23:508–18. 10.1136/bmjqs-2013-002524. 10.1136/bmjqs-2013-002524 24505110

[15] Pocock LV, Wye L, French LRM, et al. Barriers to GPs identifying patients at the end-of-life and discussions about their care: a qualitative study. Fam Pract. 2019. 10.1093/fampra/cmy135. 10.1093/fampra/cmy135 30649266

[16] Beernaert K, Deliens L, de Vleminck A (2014). Early identification of palliative care needs by family physicians: a qualitative study of barriers and facilitators from the perspective of family physicians, community nurses, and patients. Palliat Med.

[17] Claessen SJJ, Francke AL, Engels Y, et al. How do GPs identify a need for palliative care in their patients? An interview study. BMC Fam Pract. 2013;14:42. 10.1186/1471-2296-14-42. 10.1186/1471-2296-14-42 PMC3617003 23530627

[18] Harrison N, Cavers D, Campbell C, et al. Are UK primary care teams formally identifying patients for palliative care before they die? Br J Gen Pract. 2012;62(598):e344–52. 10.3399/bjgp12X641465. 10.3399/bjgp12X641465 PMC3338056 22546594

[19] Cox A, Illsley M, Knibb W, et al. The acceptability of e-technology to monitor and assess patient symptoms following palliative radiotherapy for lung cancer. Palliat Med. 2011;25(7):675–81. 10.1177/0269216311399489. 10.1177/0269216311399489 21474620

[20] Matthies LM, Taran FA, Keilmann L, et al. An electronic patient-reported outcome Ttool for the FACT-B (Functional Assessment of Cancer Therapy-Breast) questionnaire for measuring the health-related quality of life in patients with breast cancer: reliability study. J Med Internet Res. 2019;21(1):e10004. 10.2196/10004. 10.2196/10004 PMC6362389 30668517

[21] Schäffeler N, Sedelmaier J, Möhrer H, et al. Patientenautonomie und -informiertheit in der Psychoonkologie: Computerbasiertes Belastungs-Screening zur interaktiven Behandlungsplanung (ePOS-react). Psychother Psychosom Med Psychol. 2017;(67):296–303. 10.1055/s-0043-113438. 10.1055/s-0043-113438 28719921

[22] Lukasczik M, Seekatz B, Radina S, et al. Computergestütztes Screening auf Palliativbedarf bei onkologischen Patienten: Projekt zur Verbesserung der Beratung und Unterstützung von Krebspatienten und ihren Angehörigen (BUKA). Onkologe. 2016;(22):56–60. 10.1007/s00761-015-3075-1.

[23] André B, Ringdal GI, Loge JH, et al. The importance of key personnel and active management for successful implementation of computer-based technology in palliative care. Comput Inform Nurs. 2008;26(4):183–9. 10.1097/01.NCN.0000304802.00628.70. 10.1097/01.NCN.0000304802.00628.70 18600124

[24] Stukenborg GJ, Blackhall L, Harrison J, et al. Cancer patient-reported outcomes assessment using wireless touch screen tablet computers. Qual Life Res. 2014;(23):1603–7. 10.1007/s11136-013-0595-2. 10.1007/s11136-013-0595-2 24307212

[25] Maguire R, McCann L, Miller M, et al. Nurse's perceptions and experiences of using of a mobile-phone-based Advanced Symptom Management System (ASyMS) to monitor and manage chemotherapy-related toxicity. Eur J Oncol Nurs. 2008;12(4):380–6. 10.1016/j.ejon.2008.04.007. 10.1016/j.ejon.2008.04.007 18539527

[26] Basch E, Abernethy AP (2011). Supporting clinical practice decisions with real-time patient-reported outcomes. J Clin Oncol.

[27] Grol R, Wensing M, Bosch M, et al. Theories on implementation of change in healthcare. In: Grol R, Wensing M, Eccles M, Davis D, editors. Improving patient care: the implementation of change in health care. 2^nd^ ed. Oxford: Wiley; 2013. pp. 18–39. 10.1002/9781118525975.

[28] Antunes B, Harding R, Higginson IJ (2014). Implementing patient-reported outcomes measures in palliative care clinical practice: a systematic review of facilitators and barriers. Palliat Med.

[29] Meuser M, Nagel U, Bogner A, Littig B, Menz W (2009). The expert interview and changes in knowledge production. Interviewing experts.

[30] Schreier M (2012). Qualitative content analysis in practice.

[31] Kruse J (2014). Qualitative Interviewforschung: Ein integrativer Ansatz.

[32] German National Academy of Sciences Leopoldina and Union of German Academies of Sciences and Humanities. Palliative care in Germany – Perspectives for Practice and Research. 2015. https://www.akademienunion.de/fileadmin/redaktion/user_upload/Publikationen/Stellungnahmen/2015_Palliativversorgung_EN.pdf. Assessed 14 Jan 2020.

[33] O'Brien BC, Harris IB, Beckman TJ, et al. Standards for reporting qualitative research: a synthesis of recommendations. Acad Med. 2014;89(9):1245–51. 10.1097/ACM.0000000000000388. 10.1097/ACM.0000000000000388 24979285

[34] Renn O. Technik in der gesellschaftlichen Auseinandersetzung: Überblick über die Ergebnisse der Akzeptanzforschung. In: Umwelt, Wirtschaft, Gesellschaft: Wege zu einem neuen Grundverständnis; Kongreß der Landesregierung "Zukunftschancen eines Industrielandes". Stuttgart; 1986. p. 274–83. 10.18419/opus-7267. https://d-nb.info/1117555143/34. Accessed 31 July 2019.

